# *Brugia malayi* microfilariae adhere to human vascular endothelial cells in a C3-dependent manner

**DOI:** 10.1371/journal.pntd.0005592

**Published:** 2017-05-08

**Authors:** Jan-Hendrik Schroeder, David McCarthy, Tadge Szestak, Darren A. Cook, Mark J. Taylor, Alister G. Craig, Charlotte Lawson, Rachel A. Lawrence

**Affiliations:** 1 Royal Veterinary College, Department of Comparative Biomedical Sciences, Royal College Street, London, United Kingdom; 2 UCL School of Pharmacy, London, United Kingdom; 3 Liverpool School of Tropical Medicine, Pembroke Place, Liverpool, United Kingdom; University of Liverpool, UNITED KINGDOM

## Abstract

*Brugia malayi* causes the human tropical disease, lymphatic filariasis. Microfilariae (Mf) of this nematode live in the bloodstream and are ingested by a feeding mosquito vector. Interestingly, in a remarkable co-evolutionary adaptation, Mf appearance in the peripheral blood follows a circadian periodicity and reaches a peak when the mosquito is most likely to feed. For the remaining hours, the majority of Mf sequester in the lung capillaries. This circadian phenomenon has been widely reported and is likely to maximise parasite fitness and optimise transmission potential. However, the mechanism of Mf sequestration in the lungs remains largely unresolved. In this study, we demonstrate that *B*. *malayi* Mf can, directly adhere to vascular endothelial cells under static conditions and under flow conditions, they can bind at high (but not low) flow rates. High flow rates are more likely to be experienced diurnally. Furthermore, a non-periodic nematode adheres less efficiently to endothelial cells. Strikingly C3, the central component of complement, plays a crucial role in the adherence interaction. These novel results show that microfilariae have the ability to bind to endothelial cells, which may explain their sequestration in the lungs, and this binding is increased in the presence of inflammatory mediators.

## Introduction

Circadian rhythms are thought to maximise the fitness of parasites by increasing transmission, survival or reproductive capacity. Lymphatic filariasis is a major tropical disease, caused by the filarial nematodes, *Brugia malayi* and *Wuchereria bancrofti*. The circadian appearance of first-stage larvae or microfilariae (Mf) in the peripheral blood of their host is a phenomenon first observed by Patrick Manson in the late 19^th^ century and has been well described. Indeed, the synchronisation of microfilarial appearance in the blood with the peak feeding time of the local mosquito vector population in different parts of the world, suggests a co-evolutionary adaptation for maximising parasite transmission [1,2,3]. It is known that Mf, when they are not in the peripheral blood, generally sequester in the lung capillaries of the host [4,5,6]. However, there has been limited insight into the underlying mechanism of this pulmonary sequestration. Here we report that *B*. *malayi* Mf can bind to vascular endothelial cells (EC) *in vitro* and we suggest that active adherence of Mf to EC may be one mechanism by which Mf sequester in the lungs of the human host.

In a number of papers in the 1960s, (reviewed in [4]) Hawking showed that many species of filariae display periodicity and that while microfilariae have their own weak endogenous circadian rhythm, their rhythms are dominated by that of the host. So, for example, if the light:dark cycle of the host is reversed the periodicity of the microfilariae is also inverted [4]. The exact mechanism of periodicity has proved elusive, though the periodicity of particular parasites appears to be differentially effected by oxygen tension or temperature. For example, *Wuchereria bancrofti* and *Brugia malayi* microfilariae, which are normally nocturnally periodic or sub-periodic [3], fall in the peripheral blood at night both when the host breathes oxygen and when the host takes exercise. Other filarial species appear to respond to a lack of oxygen rather than a rise of oxygen [4]. Conversely, for some periodic filarial nematodes eg *Edesonfilaria malayensis* in the macaque, a rise in temperature rather than oxygen levels causes disappearance of the microfilariae from the periphery. (Even non-periodic filariae including *Litomosoides carinii* (now *sigmodontis*) respond to a temperature drop to accumulate in the peripheral blood, which is thought to enable uptake by their burrow-dwelling mite vectors). Administration of serotonin into several filarial-infected hosts also causes an immediate release of Mf from the lungs into the peripheral blood [7,8]. Interestingly, both a hypoxic environment in the lung, and administration of serotonin will cause vasoconstriction, while a rise in oxygen in the lung or exercise will result in increased ventilation and vessel dilation. This suggests that the vascular endothelium may play a critical role in Mf release [9]. Intriguingly, *B*. *malayi* Mf secrete potent vasodilators such as, prostacyclin and prostaglandin E_2_ (PGE_2_), which may allow these parasites to modulate vasculature function in order to interact with the endothelial surface [10,11].

A close association of Mf with host vascular endothelium is not without precedent as several blood-borne parasites have been shown to form an intimate host-parasite relationship with these cells. *Schistosoma mansoni* eggs adhere to EC surface molecules, ICAM-1, VCAM-1 and E-selectin via egg surface-expressed Lewis X antigen-related carbohydrates [12]. In common with *S*. *mansoni* eggs, *Plasmodium falciparum*-infected erythrocytes also bind to ICAM-1, VCAM-1 and E-selectin expressed on EC [13,14,15,16]. Furthermore, *P*. *falciparum*-infected erythrocytes ligate to the endothelial surface molecules, CD36, thrombospondin, CD31, P-selectin and complement receptor 1 (CR1), as well as, chondroitin sulphate A (CSA) and hyaluronic acid [16,17,18,19,20].

In this study, we modelled the interaction between *B*. *malayi* Mf and vascular EC *in vitro*. Notably, *B*. *malayi* Mf bind to the surface of human umbilical vein EC (HUVEC) and they do so in far greater numbers than Mf of the filarial nematode, *Litomosoides sigmodontis*. Interestingly Kwa *et al*. (1991) also noted that *B*. *malayi* microfilariae can be seen adhering to mouse endothelial cells cultured from infected athymic mice [21]. We found that presence of complement component 3 (C3) promoted *B*. *malayi* Mf adherence, and indeed, C3 was seen deposited on the surface of Mf. However, the complement receptor CR1, which binds C3b and inactivated C3b (iC3b), and CR4, which binds only iC3b, do not appear to play a role in this interaction. Several other treatments also promoted *B*. *malayi* Mf adherence to HUVEC, although to a lesser degree than C3; these included stimulation of HUVEC with IFN-γ (but not TNF-α) and treatment of HUVEC with fixatives. The presence of diethylcarbamazine (DEC), which is known to cause the immediate release of Mf from the lungs into the peripheral blood of hosts *in vivo*, did not alter Mf adherence to EC *in vitro* [7,22,23,24]. Conditions found in the sleeping host, such as the presence of melatonin, or a culture temperature of 36°C, did not influence Mf binding but Mf were not able to bind endothelial cells either when they were exsheathed or in a hypoxic environment. Interestingly, in an *in vitro* EC culture system, high flow rates, similar to those found in the host during the day, enabled Mf binding, while low flow rates, which may be found at night, did not result in Mf adherence. This study provides further clues to the mechanistic explanation for the retention of Mf in host non-peripheral blood vessels during their circadian periodicity.

## Materials and methods

### Ethics statement

Ethical approval was obtained from the East London Local Research Ethics Committee to collect human umbilical cords from mothers at the Royal London Hospital and blood from healthy donors. All study participants provided written informed consent. Parasite maintenance in animals and infection of animals was in accordance with the Home Office project licence (PPL 70/7243), which was approved by the Home Office under the Animal Scientific Procedures Act (1986), UK. The project was approved by the local Ethical Review Committee at the Royal Veterinary College, London UK.

### Cell culture

HUVEC were isolated from human umbilical cords, obtained from the Royal London Hospital, using a modified method [25,26]. In all experiments, HUVEC were used at passage 5, and the cell morphology was confirmed by phase contrast microscopy. HUVEC were cultured in HUVEC medium (Medium 199 supplemented with 150U/ml penicillin, 150U/ml streptomycin, 2mM L-glutamine (Sigma-Aldrich Ltd, Gillingham, Dorset, UK), 20% heat-inactivated FBS (GE Healthcare Europe GmbH, Vienna, Austria), 1U/ml heparin and 0.03mg/ml endothelial cell growth supplement from bovine neural tissue (Sigma-Aldrich Ltd). HEK 293T cells and the transformed human umbilical vein endothelial cell line, EaHy926, were maintained in DMEM (Sigma-Aldrich Ltd) supplemented with 10%FBS, 150U/ml penicillin and 150U/ml streptomycin. THP-1 cells were cultured in RPMI-1640 (Sigma-Aldrich Ltd) supplemented with 10%FBS, 50μM β-mercaptoethanol, 150U/ml penicillin and 150U/ml streptomycin.

### Preparation of human serum

Blood was collected from young healthy donors into tubes without anticoagulant, at around midday. After 3 hours at room temperature, serum was harvested following centrifugation at 2,057xg for 20 min. In several experiments, serum was heat-inactivated at 56°C for one hour in a water bath.

### *B*. *malayi* and *Litomosoides sigmodontis* microfilariae

*B*. *malayi* and *L*. *sigmodontis* Mf were obtained by peritoneal lavage with RPMI-1640 from infected gerbils (*Meriones unguiculatus*). (*B*. *malayi* were the strain from TRS Laboratories, Athens, Georgia, USA and Liverpool School of Tropical Medicine and *L*. *sigmodontis* from Prof Judi Allen, University of Edinburgh). Mf were isolated by centrifugation over lymphocyte separation medium (MP Biomedicals, USA) [27]. *B*. *malayi* microfilariae were exsheathed by incubating them for one to three hours in 20 mM CaCl_2_ in Earls phosphate-free balanced salt solution. Approximately 70% of Mf lost their sheaths after one hour, whereas 97% had exsheathed by 3 hours and they appeared highly motile and viable. In some experiments, 250,000 Mf were injected i.v. into groups of five 6–8 week old male BALB/c mice (Harlan UK Ltd, Bicester, UK). Mice were tail bled at intervals at 11a.m., and the Mf obtained in 50μl of blood were counted following red blood cell lysis. Mice were anaesthetised i.m. with ketamine-xylazine.

### Culturing of *B*. *malayi* Mf with adherent cells

Mf and HUVEC were co-cultured throughout this study in complete Medium 199 and RPMI (1:1) plus serum and 1% glucose (Sigma-Aldrich Ltd). Unless otherwise indicated, 20% FBS was used. HUVEC were cultured in HUVEC medium for 60 hours and thereafter the medium was replaced with co-culture medium containing 125,000 *B*. *malayi* Mf. In experiments using HEK 293T and EaHy926, DMEM replaced the use of Medium 199 while in THP-1 experiments RPMI-1640 was used in place of Medium 199. All cell monolayers were fully confluent at the time of Mf co-culture. THP-1 and HUVEC were seeded for 48 hours and HEK 293T for 24 hours prior to co-culture with Mf. THP-1 are a monocytic cell line, and they are differentiated into macrophages using PMA (Sigma-Aldrich Ltd). THP-1 were stimulated with PMA (50ng/ml) 48 hours prior to adding Mf to the culture. Following co-culture, non-adhering Mf were washed off with pre-warmed medium and adherent Mf were counted using phase contrast microscopy. Further experiments were performed using a VenaFlux semi-automated microfluidic system to study Mf adhesion under shear flow conditions that are more physiologically relevant to *in vivo* blood flow rates [28]. The microfluidic system has 8-channels designed for growth of human endothelial cells such that cells are continuously fed during the experiment and parameters can be adjusted and monitored using VenaFlux software (Cellix Ltd, Dublin, Ireland). Briefly, Vena8 Endothelial+ biochip channels (Cellix Ltd) were coated with 12μl of 100μg/ml fibronectin and incubated in a humidified petri-dish at 4°C overnight. HUVEC were seeded in a chamber 30 minutes prior to performing the assay. Once the cells formed a confluent monolayer in the channels, they were exposed to Mf (10,000/ml) at 0.1–1.0 dyne/cm^2^. The ability of Mf to adhere to HUVEC under flow conditions was visualised using the VenaFlux software.

### HUVEC co-culturing with Mf in different conditions

In some experiments, HUVEC were co-cultured with Mf in medium supplemented with different concentrations and types of serum or other additional additives. These included heat-inactivated or non-heat inactivated human serum, C3-depleted human serum, 250μg/ml mannan, 2.5μM DEC or 1nM melatonin (Sigma-Aldrich Ltd. The melatonin concentration used in this experiment corresponded approximately to the concentration of melatonin observed in human plasma at early night [29]. In some experiments HUVEC were stimulated with 10ng/ml human IFN-γ (Immuno-Contact, USA) for 24-48h or 20ng/ml human TNF-α (Insight Biotechnology Ltd., Wembley, UK) for 18h prior to co-culture with Mf. In other experiments, cell culture plates containing endothelial cells were placed inside a sealed plastic container that also contained a GasPak TM EZ Campy Container System Sachet (BENEX Ltd., Maryland, USA) to achieve a hypoxic environment. (Each sachet contains inorganic carbonate, activated carbon, ascorbic acid and water, which reduces the oxygen concentration within the container to 6–16%). The number of Mf adhering after 24 h co-culture was counted. Both cell and Mf viability, and Mf motility was unaltered after 24 hours in the hypoxic environment.

### HUVEC incubation with blocking antibodies

In several experiments blocking antibodies were used against HUVEC. The monoclonal antibody hybridoma cells, HB32 (mouse anti-I-E^k^), W6/32 (mouse anti-human HLA-A, B, C), L243 (mouse anti-human HLA-DRα), 6.5B5 (mouse anti-human ICAM-1) and DA6.231 (mouse anti-human HLA-DRβ) were grown *in vitro*. Supernatant was harvested, and antibody was purified over protein G Sepharose. Working dilutions of antibody were determined by staining HUVEC pre-stimulated with 10ng/ml human IFN-γ (Immuno-Contact, USA) for 48 hours. HUVEC were incubated for 30 minutes at the optimal working dilution. Mouse anti-I-E^k^ (IgG2a; 14-4-4S) was used as an isotype control antibody for W6/32 and L243 clone antibodies, while mouse anti-IgG1 (TNP-specific) (BD Pharmingen, Oxford, UK) was used as an isotype control for D6.231 and 6.5B5 clone antibodies. Mouse anti-human CR1 (3D9 clone) antibody has previously been used to block the ligation of C3b to CR1 [30]. HUVEC were incubated with 3D9 antibodies for 45 minutes at the optimal working dilution. Mouse anti-human IgG1 (TNP-specific) was used as an isotype control at the same concentration. After incubation with antibody, the cells were washed three times with HUVEC medium. The surface blocking of markers was performed immediately prior to co-culture with Mf.

### Fixing and killing of HUVEC

HUVEC were treated with 7.8μM sodium azide (Sigma-Aldrich Ltd.) for 2h at 37°C to kill them. Cells were then washed 5 times with pre-warmed PBS to remove all traces of sodium azide before co-culture with Mf. For fixation, HUVEC were treated with 4% pre-warmed formaldehyde or pre-warmed 2% formaldehyde plus 2.5% glutaraldehyde and 25mM L-lysine (Sigma-Aldrich Ltd.) in PBS for 10 minutes before washing with PBS prior to co-culture with Mf.

### Scanning electron microscopy (SEM)

*B*. *malayi* Mf that had bound to HUVEC were fixed in 4% paraformaldehyde for 10 minutes at room temperature. The samples were dehydrated in 50% ethanol for 5 minutes, followed by 100% ethanol for 2 x 5 minutes. Then samples were adhered onto an SEM stub (Taab Laboratories Equipment Ltd., Reading, UK) using carbon adhesive discs (Agar Scientific Ltd, Stansted, UK). Prior to viewing, the prepared sample was coated with 20nm gold in a Quorum Q150T sputter coater (Quorum, Technologies Ltd, Laughton, UK). Digital images of the samples were captured under an FEI Quanta 200F SEM (FEI, Eindhoven, The Netherlands). The structural features of *B*. *malayi* Mf were characterized according to a previous publication [31].

### Flow cytometry

For flow cytometry, antibodies were either used at the optimal working dilution as outlined above or according to the manufacturer`s instructions (mouse anti-human CD11c (Serotec)). Goat anti-mouse FITC conjugated antibodies (Sigma-Aldrich Ltd.) were used as a secondary antibody with unconjugated primary antibodies. Data were acquired using a FACS Canto II (BD Oxfordshire UK) and analysed with FlowJo software (Tree Star Incorporation, Ashland, Oregon, USA).

### Statistics

Student’s t-test for paired data and ANOVA was used for statistical analyses. A *p* value < 0.05 was considered to be statistically significant. Data are presented as mean ± standard deviation.

## Results

### *B*. *malayi* Mf adhere to vascular endothelial cells

**Fig 1:**
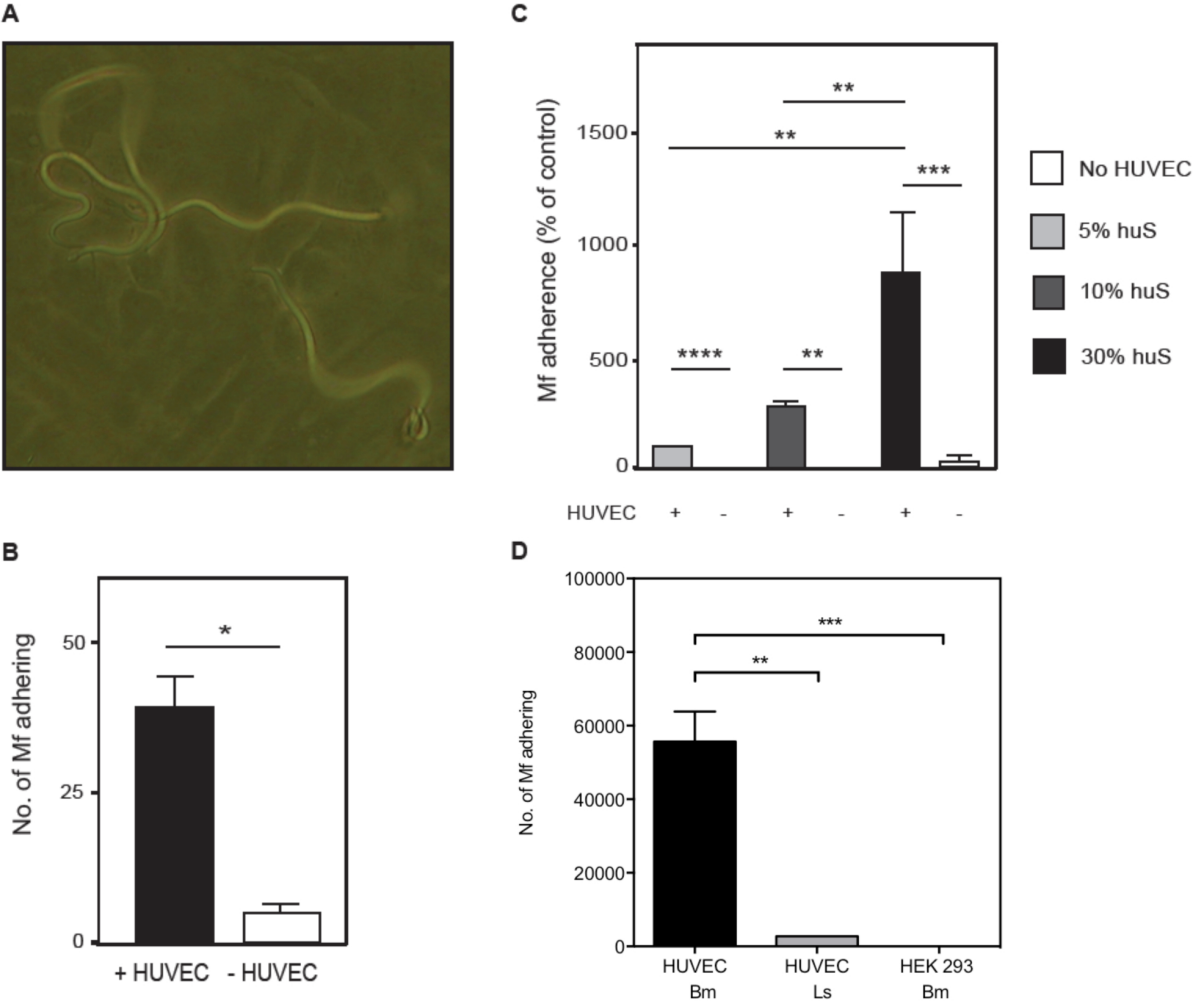
*B*. *malayi* Mf adhere to HUVEC with one or both ends. Mf were co-cultured with HUVEC, HEK293 or no cells for 24 hours. (A) Side-to-side movement of *B*. *malayi* Mf bound to HUVEC (magnification 20 x). (B) The number of Mf adhering to HUVEC in medium supplemented with 20% heat-inactivated FBS or (C) medium supplemented with 5, 10 or 30% intact human serum (huS) was determined. (D) The number of *B*. *malayi* (Bm) adhering to HUVEC and HEK293 cells and the number of *L*. *sigmodontis* (Ls) Mf adhering to HUVEC. Data are shown as the mean ± standard deviation of three independent experiments and Students t-test was performed to analyse statistical significance where * p<0.05, ** p<0.01, *** p<0.001, **** p<0.0001.

Intriguingly, when *B*. *malayi* Mf were incubated with HUVEC *in vitro*, a proportion of Mf were seen to attach to the cell surface. Mf used one end, and sometimes both ends simultaneously, to interact with the cell monolayer (Fig 1A, S1 Supporting Information). If *B*. *malayi* Mf are damaged it is known that they bind to plastic tissue culture surfaces. To ensure that Mf were binding specifically to HUVEC, the number of Mf adhering in the presence or absence of HUVEC was compared (Fig 1B and 1C). Few Mf bound to the gelatine-coated flask alone, while in the presence of HUVEC a significant number of *B*. *malayi* Mf were seen attached to the cell surface. Strikingly, Mf also adhered *in vitro* to HUVEC under high flow conditions (1 dyne/cm^2^), but not during slow flow conditions (0.1 dyne/cm^2^) (S2–S4 Supporting Information). In S4 Supporting Information, a Mf can be seen attempting to adhere under high flow with the anterior end. Furthermore, in order to determine whether Mf bind only to HUVEC or whether they are able to bind other adherent cell types, the ability of Mf to bind HUVEC, HEK 293T (a human embryonic kidney cell line) and PMA-stimulated THP-1 (a human monocytic cell line), was compared (Fig 1D, S5 Supporting Information). *B*. *malayi* Mf bound to HUVEC but not significantly to HEK 293T. *B*. *malayi* Mf also bound to activated THP-1 adhering in a monolayer though this could not be accurately enumerated as some THP-1 cells released from the plastic tissue culture surface and adhered to the surface of the Mf (S5 Supporting Information).

To gain insight into whether the adherence of *B*. *malayi* Mf to HUVEC is a common characteristic of filarial Mf, the ability of *L*. *sigmodontis* Mf to adhere to HUVEC was investigated. Strikingly, only low numbers of Mf of this species bound to HUVEC (Fig 1D and S6 Supporting Information).

**Fig 2:**
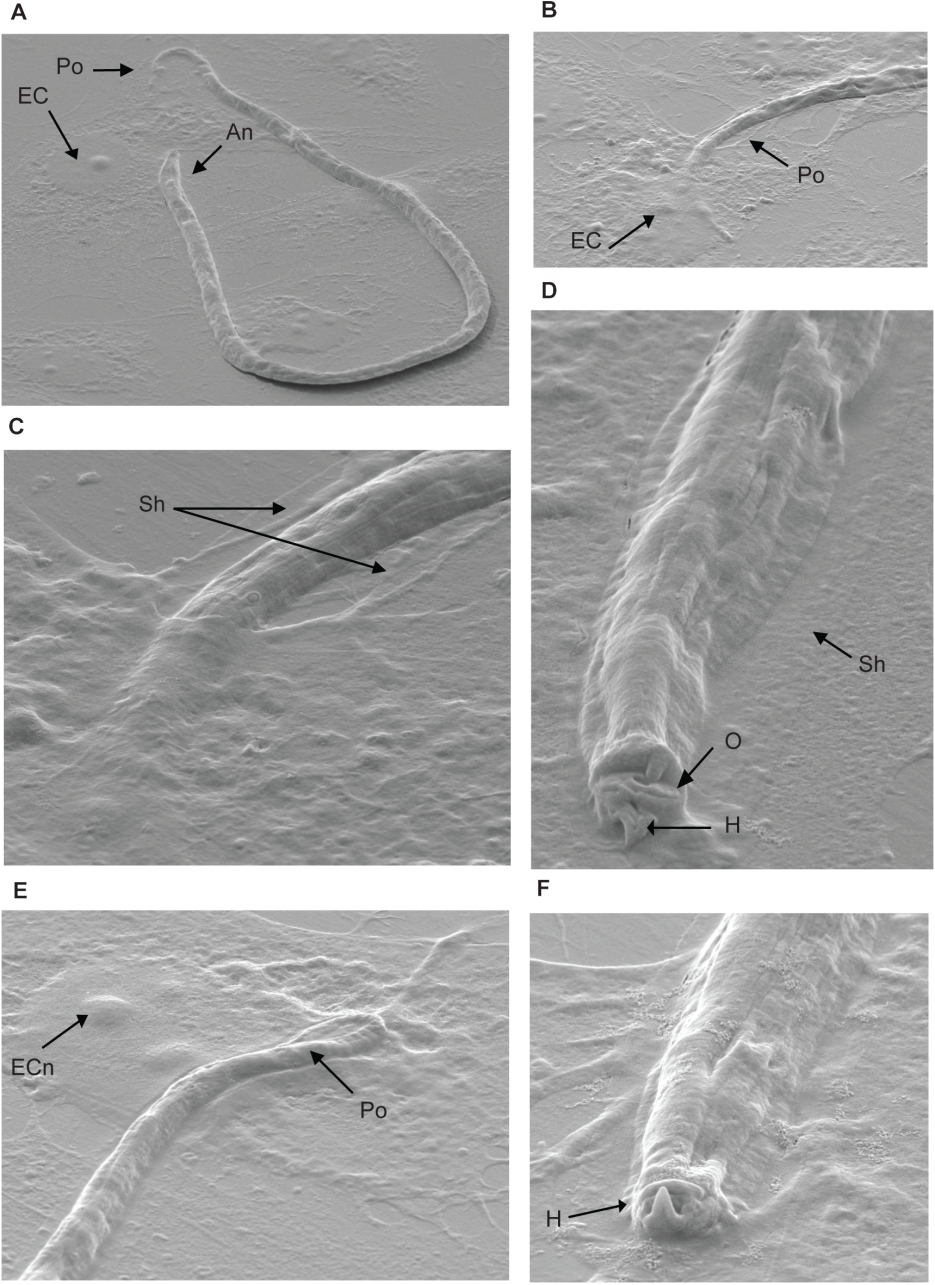
*B*. *malayi* Mf adhere to HUVEC. *B*. *malayi* Mf were co-cultured with HUVEC in the presence of 10% intact human serum for 24 hours. Mf binding to HUVEC were examined by scanning electron microscopy. EC, endothelial cell; An, anterior end; Po posterior end; Sh, microfilarial sheath; H, hook; O, oral ring; ECn EC nucleus (magnification: 2000 x (A), 4000 x (B), 6000 x (C), 16000 x (D), 15000 x (E), 20000 x (F)).

Scanning electron microscopy (SEM) was performed to study the binding of *B*. *malayi* Mf to vascular EC in more detail (Fig 2). Several structures previously seen at the anterior end of Mf, such as the hook, ocular mouth and spines were identified [31,32]. SEM also revealed that Mf ligated to the cell surface with either the anterior or posterior ends, and in some cases, they appeared to have burrowed beneath or inside the cell.

### Hypoxia, but not melatonin, night body temperature and diethylcarbamazine alters Mf adherence to vascular endothelial cells *in vitro*

**Fig 3:**
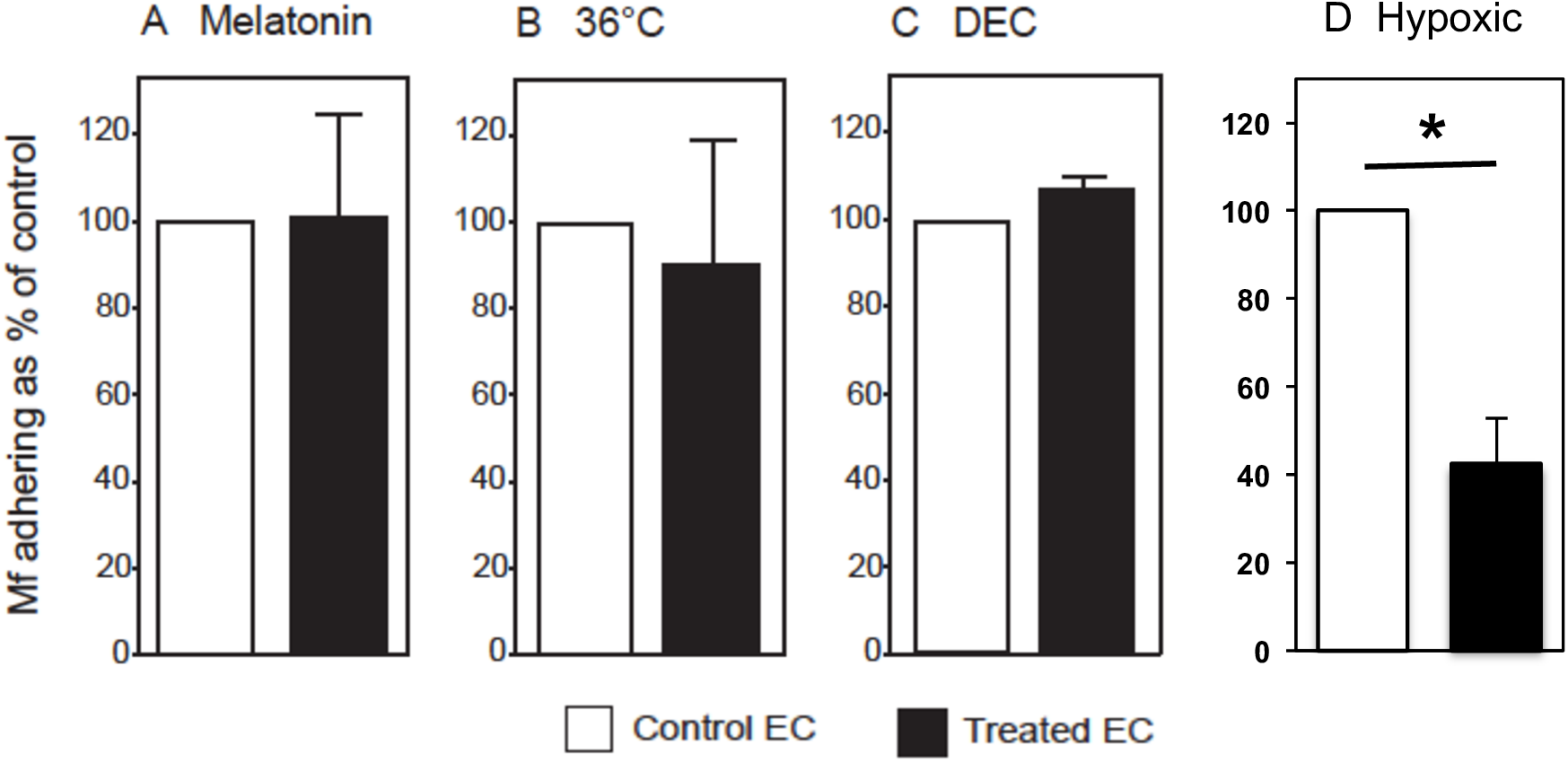
Hypoxia but not melatonin, 36°C temperature or DEC alters *B*. *malayi* Mf adherence to HUVEC. *B*. *malayi* Mf were co-cultured with HUVEC for 24 hours in medium supplemented with or without 1nM melatonin (A) or 2.5μM DEC (C) and the number of Mf adhering were counted. In some experiments HUVEC and Mf were co-cultured at 36°C or 37°C (B). In other experiments *B*. *malayi* Mf were co-cultured with HUVEC in hypoxic conditions (D). Data are shown as the mean ± standard deviation of three independent experiments and Students t-test was performed to analyse statistical significance where * indicates p <0.05.

One possible signal for driving filarial nematode periodicity may be the secretion of melatonin in the host at the beginning of the sleep cycle. Since *B*. *malayi* is a nocturnally periodic nematode, melatonin could act as a trigger for the release of Mf from the lung capillaries into the peripheral blood. To investigate whether melatonin negatively affects the level of Mf adherence to HUVEC, Mf and HUVEC were co-cultured in the presence of 1nM melatonin. This concentration of melatonin corresponds to the amount observed in humans in the early hours of the morning [29]. However, the presence of melatonin did not alter the number of Mf binding to vascular EC *in vitro* in static culture (Fig 3A).

Another possible trigger for Mf release into the peripheral blood is the drop in human body temperature, to approximately 36°C, that occurs during sleep. Indeed, Hawking *et al*. (1968), found that although in some hosts *B*. *malayi* Mf did not respond to temperature fluctuations [4], in primates lowered body temperature correlated with raised *B*. *malayi* Mf counts in the peripheral blood [7]. To try to model this *in vitro*, Mf and HUVEC were incubated together at either 36°C or 37°C, however, no significant differences in the number of adhering Mf were found between the two temperatures in static culture (Fig 3B).

As DEC causes the release of Mf into the peripheral blood circulation *in vivo*, we investigated whether 2.5μM DEC inhibits the adherence of Mf to HUVEC *in vitro*. After 24 hours, the number of Mf adhering to HUVEC did not differ in the presence or absence of DEC (Fig 3C). Thus, neither melatonin, DEC nor a drop in temperature has a direct effect upon microfilarial binding to HUVEC *in vitro*.

*In vivo* studies have suggested that differences in oxygen tension can release Mf into the peripheral blood [4]. When EC were maintained in a hypoxic environment we found that significantly fewer Mf adhered to the EC surface (p<0.05) (Fig 3D).

**Fig 4:**
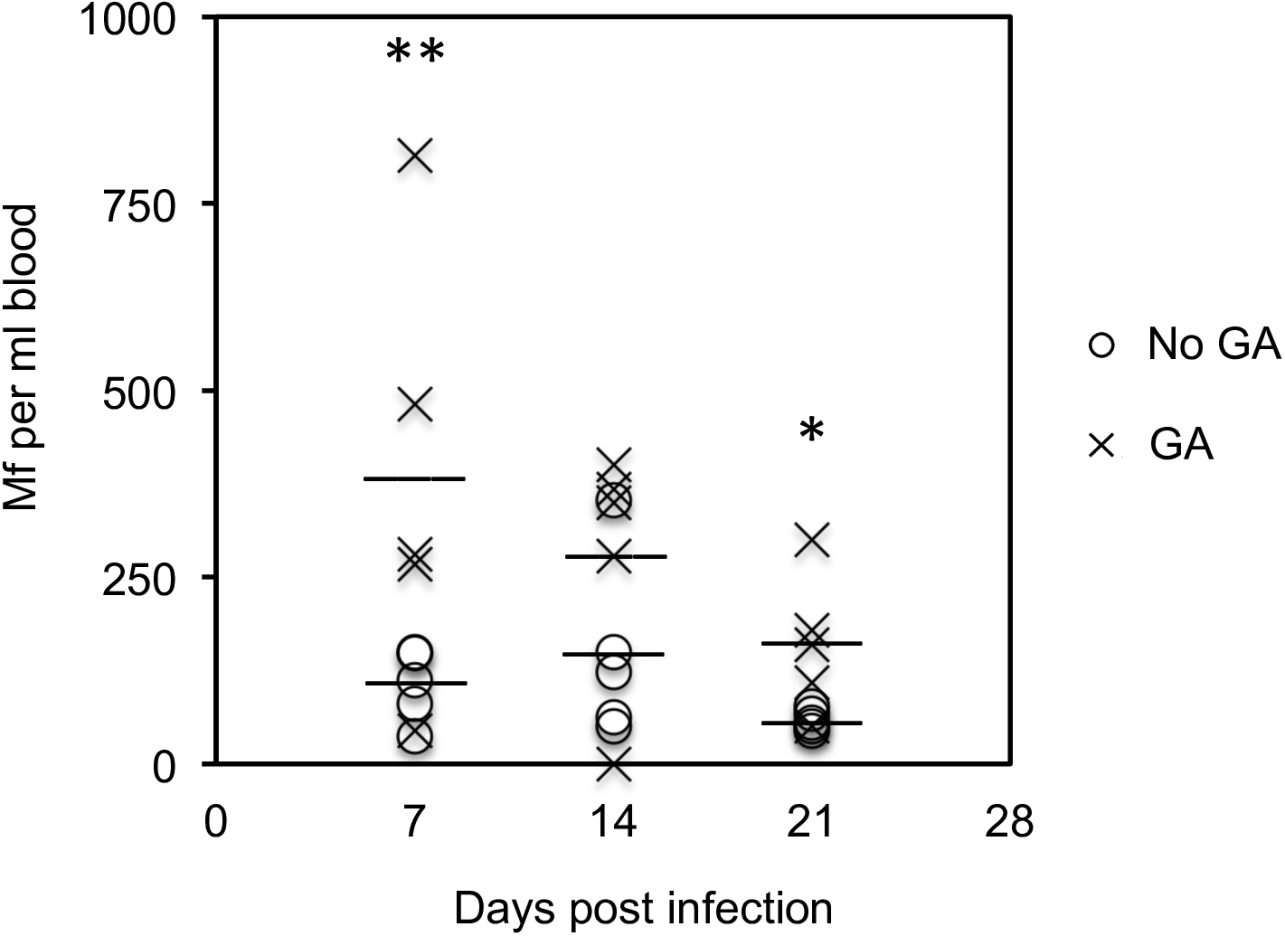
*B*. *malayi* Mf counts in mice under general anaesthesia. Groups of 5 individual BALB/c mice were injected with B. malayi Mf iv and bled at 11am at intervals. Mice were either left unanaesthetised (No GA) or were given general anaesthetic (GA) before bleeding from the tail vein. ANOVA was performed to measure statistical significances where * indicates p <0.05 and ** indicate p<0.01.

To confirm whether the *B*. *malayi* Mf strain from TRS labs still varies in number in the host when it is asleep, Mf were counted in the blood when the host was awake or when it was under general anaesthesia over a three week period. Mf counts were nearly all higher in mice that were under general anaesthesia, and despite variable Mf counts, this was significantly different at day 7 and 21 post infection (Fig 4).

### *B*. *malayi* Mf have enhanced adherence to fixed but not dead HUVEC

**Fig 5:**
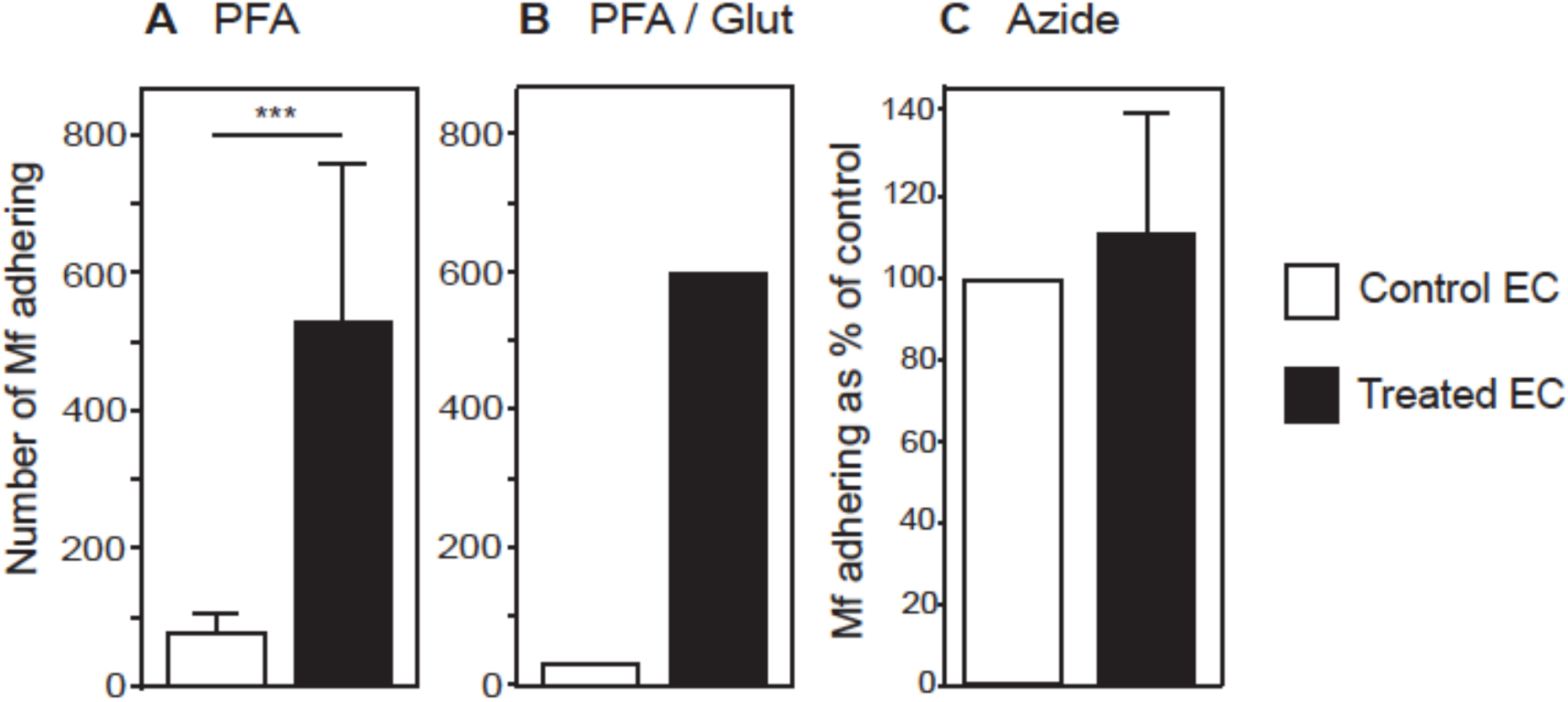
*B*. *malayi* Mf preferentially adhere to fixed HUVEC. HUVEC were fixed with 4% formaldehyde (A), 2% formaldehyde plus 2.5% glutaraldehyde and 25 mM lysine (B) or 7.8 μM sodium azide (C) prior to co-culture with *B*. *malayi* Mf for 24 hours and the number of adhering Mf was determined. Data are shown as the mean ± standard deviation of three independent experiments and Students t-test was performed to analyse statistical significance where *** p<0.001.

To investigate whether Mf adherence to HUVEC is a simple process involving EC surface molecules or a process requiring EC activity, Mf were incubated with either untreated HUVEC or HUVEC treated with fixative prior to co-culture. The number of Mf bound to HUVEC was increased by at least 10-fold, when HUVEC had been fixed with either PFA (p<0.001) or PFA-glutaraldehyde (Fig 5A and 5B). As fixed cells are not alive, we examined whether Mf adherence is simply increased following cell death. HUVEC were killed by azide treatment prior to Mf co-culture, yet still adhered in a confluent monolayer to the flask. However, the level of Mf binding to live and dead HUVEC was not significantly different (Fig 5C). These experiments suggest that Mf adhere to a surface molecule(s) expressed by vascular EC that is unmasked or cross-linked following fixation.

### HUVEC stimulation with IFN-γ, but not TNF-α, enhances Mf adherence

To further investigate the potential cell surface molecules to which Mf may adhere, HUVEC were stimulated with IFN-γ or TNF-α, both of which are known to up-regulate a broad range of EC surface molecules [33,34,35,36,37,38,39]. Mf are also known to induce a pro-inflammatory response, and this may occur in their immediate vascular environment in the host [40].

**Fig 6:**
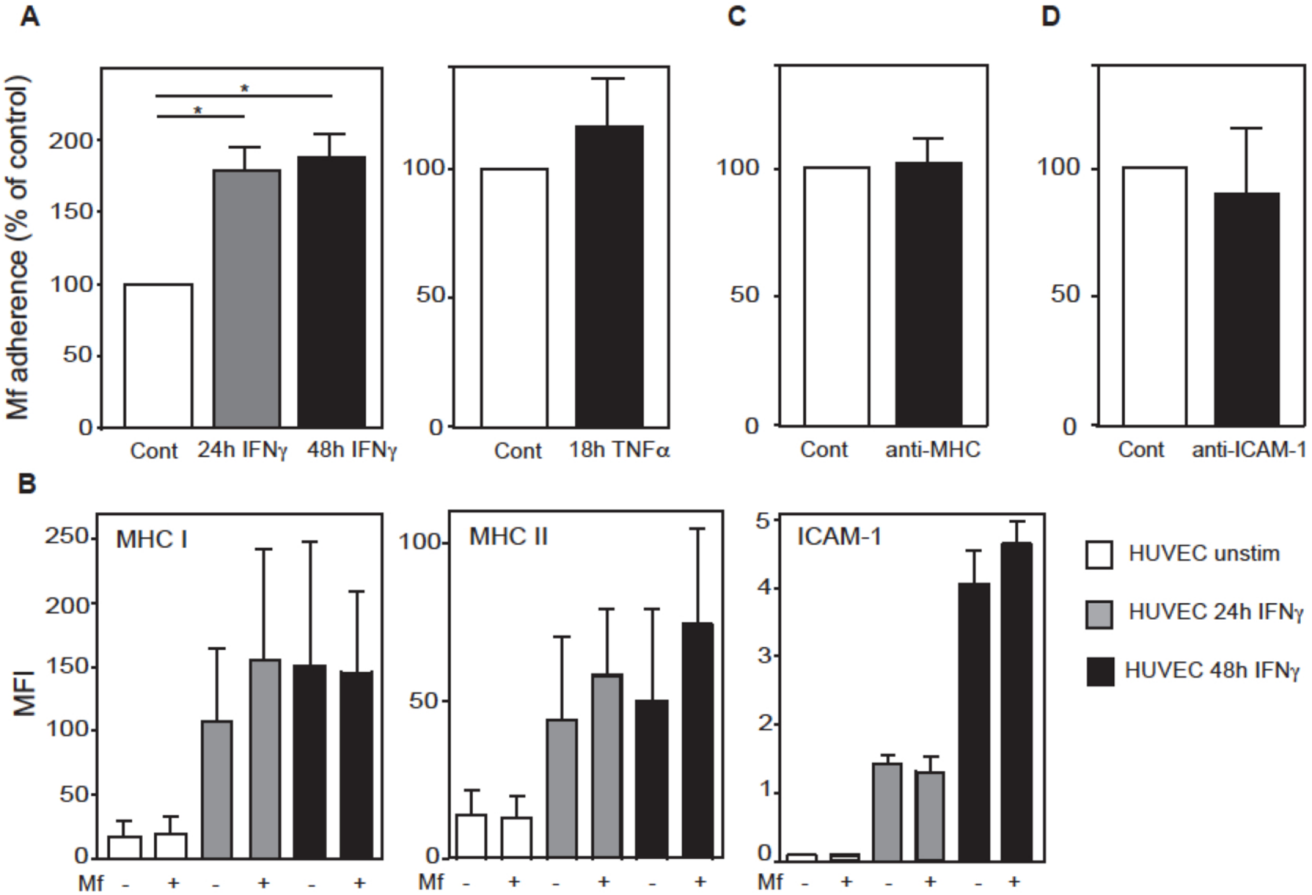
IFN-γ stimulation of HUVEC enhances Mf adherence. HUVEC were stimulated with IFN-γ (10 ng/ ml) for 24 or 48 hours (A-B) or with TNF-α (20 ng/ ml) for 18 hours (A) prior to co-culture with *B*. *malayi* Mf. Adhering Mf were counted after a further 24 hours. (B) IFN-γ-stimulated HUVEC (as in A) were analysed by FACS for MHC class I, MHC class II or ICAM-1 expression. (C) HUVEC stimulated with IFN-γ for 48 hours prior to co-culture with Mf were incubated with blocking antibodies. Anti-MHC antibodies specific for HLA-A, -B and–C, HLA-DRα and HLA-DRβ, or (D) anti-ICAM-1 or respective isotype control antibodies were incubated with IFN-γ stimulated HUVEC for 30 minutes immediately prior to co-culture with Mf. Data are shown as the mean ± standard deviation of three independent experiments and Students t-test was performed to analyse statistical significance where * p<0.05.

When HUVEC were stimulated with IFN-γ for 24 or 48 hours prior to Mf co-culture, the number of adhering Mf was significantly upregulated (p<0.05) (Fig 6A). In contrast, TNF-α stimulation of HUVEC did not alter the efficacy of Mf binding (p>0.05) (Fig 6A). IFN-γ is known to up-regulate the surface expression of MHC class I, MHC-II and ICAM-1 on vascular EC, and this was confirmed in this study (Fig 5B). Interestingly, the presence of *B*. *malayi* Mf did not alter the level of expression of any of these surface markers in the presence or absence of IFN-γ (Fig 6B). To investigate whether Mf adhere to one or more of these IFN-γ-induced endothelial surface markers, specific monoclonal antibodies were used to block potential binding sites of MHC-I and MHC-II or ICAM-1 prior to co-culture with Mf. Blocking both MHC-I and MHC-II (Fig 6C), or ICAM-1 alone did not alter the level of Mf adherence to HUVEC (Fig 6D).

### Heat-inactivation of serum partially abrogates Mf adherence to vascular EC

**Fig 7:**
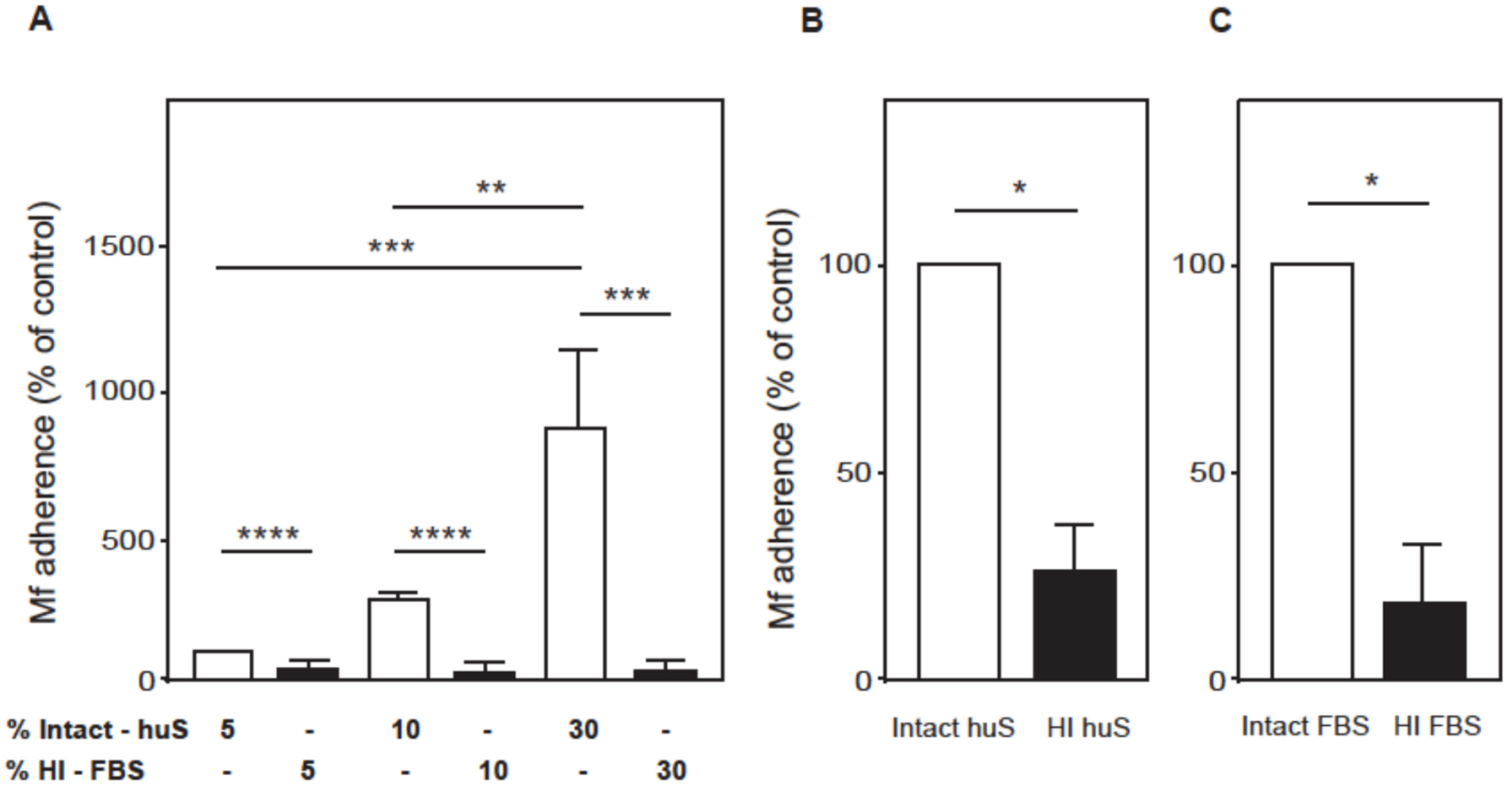
Heat-inactivation of serum greatly reduces *B*. *malayi* Mf adherence to HUVEC. HUVEC were co-cultured with *B*. *malayi* Mf for 24 hours. The medium was supplemented with, either 5%, 10% or 30% intact human serum (huS) or heat-inactivated (HI) FBS (A). In other experiments the medium was supplemented with 10% intact or heat-inactivated human serum (B) or FBS (C). Non-adhering Mf were removed and remaining Mf were counted using phase microscopy. Data are shown as the mean ± standard deviation of three independent experiments and Students t-test was performed to analyse statistical significance where * p<0.05, ** p<0.01, *** p<0.001, **** p<0.0001.

Although *B*. *malayi* Mf adhere to HUVEC *in vitro*, the actual number of Mf adhering was low when heat-inactivated FBS was used as the serum supplement in the medium (Fig 1B). *In vivo*, Mf of this nocturnally sub-periodic strain of filarial nematode are found in low numbers in the peripheral blood during the day, but show a marked nocturnal rise [3]. Therefore, if binding of Mf to EC is a mechanism by which Mf sequester in the lung capillaries during the day, the actual proportion of Mf able to bind EC would be expected to be much higher. Thus, we decided to follow a more physiological approach by co-culturing HUVEC and Mf in the presence of human serum that had not been heat-inactivated. Strikingly, increasing concentrations of intact human serum, but not heat-inactivated FBS, correlated with significantly enhanced Mf binding (Fig 7A). Addition of 5–30% intact serum compared to 5–30% heat-inactivated serum had no effect on cell viability. In further experiments we compared the binding of Mf in the presence of intact and heat-inactivated human serum, and, intact and heat-inactivated FBS. Interestingly, Mf binding was significantly decreased in the presence of heat-inactivated human or FBS compared to intact serum (p<0.05) (Fig 7B and 7C). Moreover, intact human serum or intact FBS equally promoted the adherence of Mf to HUVEC (S7 Supporting Information).

### C3 plays a central role in the adherence of *B*. *malayi* Mf to HUVEC

**Fig 8:**
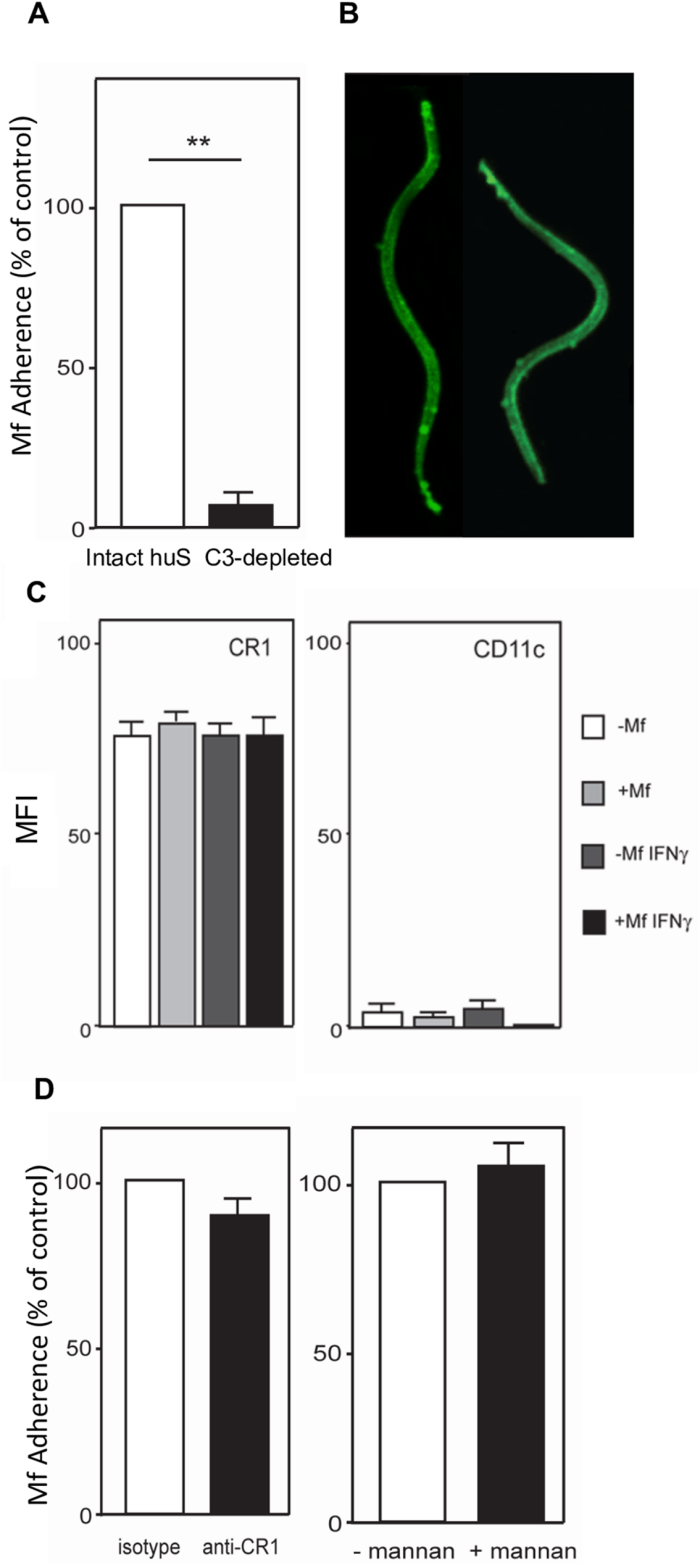
Presence of C3 enhances Mf adherence. Level of *B*. *malayi* Mf adherence to HUVEC after co-culturing for 24 hours (A) in the presence of 10% intact human serum or 10% human serum depleted of C3. (B) Mf were incubated in intact sera and C3 deposition was detected using FITC conjugated anti-C3 Ab. (C) HUVEC were stimulated with or without IFN-γ (10 ng/ml) for 48 hours prior to a 24 hour incubation with Mf. HUVEC were then analysed by FACS for surface expression of CR1 or CD11c. Data show mean fluorescence intensity (MFI) of CR1 and CD11c. (D) HUVEC were pre-treated either with an isotype control antibody or with anti-CR1 for 45 minutes immediately prior to Mf co-culture for 24 hours. In another experiment, HUVEC were pre-treated for 45 minutes in the presence or absence of 250μg/ml mannan prior to Mf co-culture. Mf binding was counted after 24 hours of co-culture. Data are shown as the mean ± standard deviation of three independent experiments and Students t-test was used to analyse statistical significance where ** p<0.01.

Complement proteins are well known to be heat-labile [39]. To identify factors that promote Mf adherence, the role of C3, the central complement protein, was further analysed. HUVEC were incubated with Mf in the presence of either C3-depleted human serum or untreated human serum. Significantly, the number of Mf bound to HUVEC after 24 hours was greatly reduced in medium supplemented with C3-depleted serum compared to untreated serum (p<0.01) (Fig 8A). In the presence of untreated serum, the C3 cleavage products, C3b and/or iC3b, are likely to bind to the microfilarial sheath and, may in turn, function as ligands for any C3 receptors expressed on HUVEC. Indeed, C3 depositions can be detected on the surface of Mf cultured with intact untreated serum (Fig 8B). Close inspection of these depositions suggests that they are particularly concentrated at the anterior and posterior ends of Mf.

HUVEC express the complement receptors CR1 and CR4. CR1 binds to C3b and iC3b, whereas CR4 binds solely to iC3b. Both receptors could potentially play a role in Mf adherence. To investigate this, the levels of CR1 and the CR4 subunit, CD11c, were examined on the surface of HUVEC following IFN-γ stimulation and subsequent incubation with or without Mf (Fig 8C). Expression of CR1 and CD11c was not altered by either Mf or IFN-γ. In contrast to CR1, HUVEC surface expression of CD11c was relatively low. Therefore, it appeared more likely that Mf adherence occurred via the C3b:CR1 or iC3b:CR1 axes. To test this hypothesis, CR1 expressed on the surface of HUVEC was blocked using a monoclonal antibody prior to co-culture with *B*. *malayi* Mf (Fig 8D). However, blockade of CR1 had no effect on Mf adherence (Fig 8D).

The lectin pathway is one of the three complement pathways that results in the activation of C3. To investigate whether the MBL pathway plays a role in the adherence of Mf to HUVEC, a co-culture was performed in the presence of excess mannan, which inhibits MBL activity. Mf adherence to HUVEC did not alter upon treatment with mannan (Fig 8D).

### Exsheathment of *B*. *malayi* Mf impairs adherence to HUVEC

**Fig 9:**
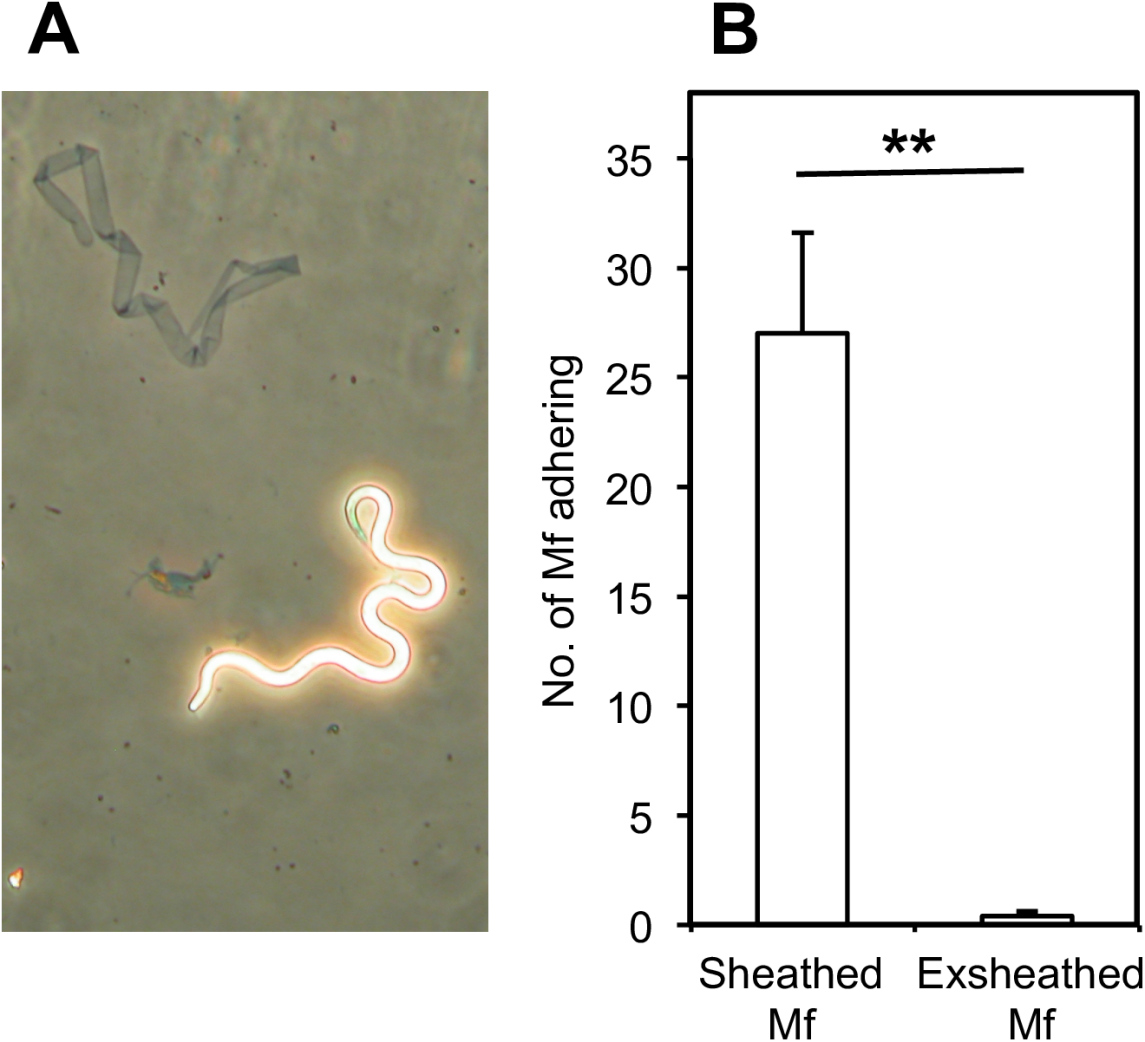
Exsheathing impairs *B*. *malayi* Mf adherence to EaHy926 cells. Untreated or exsheathed *B*. *malayi* Mf were co-cultured with EaHy926 for 24 hours, and the number of Mf adhering were counted. The success of the exsheathing procedure was confirmed by phase microscopy (A). Data are shown as the mean ± standard deviation of three independent experiments and Students t-test was performed to analyse statistical significance (B).

As complement is known to attach to the sheath of *B*. *malayi* Mf [41], Mf were exsheathed in order to investigate whether exsheathed Mf can still adhere to EC. 20 mM CaCl_2_ treatment promoted *B*. *malayi* Mf exsheathment (Fig 9A). Untreated or exsheathed Mf were co-cultured with EaHy926 cells and adherent worms were counted after 24h. In contrast to the untreated worms, exsheathed Mf showed almost no binding to the cell layer (p<0.01).

## Discussion

Circadian periodicity of Mf is a phenomenon that has been widely reported for more than a century. It is thought that Mf periodicity maximises parasite fitness by increasing the chance of transmission. In parts of the world where the predominant mosquito vector is active at night, Mf of *B*. *malayi* and *W*. *bancrofti* appear in the peripheral blood in the early hours of the morning (nocturnally periodic strains). In endemic areas, such as the South Pacific islands, where the main vector is day-biting, *W*. *bancrofti* Mf are diurnally periodic [3]. For the remainder of the day, Mf are preferentially found in the lungs of the host [4,6]. The mechanism by which Mf sequester in the lungs and are released into the peripheral blood is largely unresolved.

Most labs worldwide now use the strain of *B*. *malayi* from TRS labs that was originally nocturnally sub-periodic (meaning that while it displayed nocturnal periodicity it could still be found in the peripheral blood in low numbers during the day). This strain was later also found to be sub-periodic in mice [42,43]. There are many records showing that filarial parasites retain their periodicity in diverse hosts and *B*. *malayi* has been shown to retain periodicity in rhesus monkeys, cotton rats, mice, gerbils and cats [5,44,45,46,47,48]. Furthermore, parasites derived from nematodes maintained in the peritoneal cavity of gerbils demonstrated periodicity in cats and mice [5,44]. The parasite strain we have used is therefore likely to have retained an ability to sequester in the lung, and indeed, mice infected iv with *B*. *malayi* Mf had significantly more Mf in the peripheral blood in mice under general anaesthesia than those that were not anesthetised.

Interestingly, we found that Mf adhere to EC in static conditions, and they can also adhere to EC under high, but not low, flow rate conditions. This interaction could provide a potential mechanism for sequestration of Mf in the lung capillaries *in vivo*. In contrast, Mf of the rodent filarial nematode, *L*. *sigmodontis*, adhered to EC only in low numbers. Strikingly, this rodent nematode is non-periodic *in vivo*.

*B*. *malayi* Mf did not adhere to an embryonic kidney cell line (HEK 293T), while there was some binding of Mf to the monocytic cell line. Interestingly, adherence of *B*. *malayi* Mf to EC appeared to be with either the anterior and/or the posterior end of the Mf while the monocytic cells were more often attached to any part of the Mf surface in a manner reminiscent of their role as immunoprotective cells. A previous observation of Mf sticking to EC with one end has been made in mouse EC isolated *ex vivo* from an infected athymic mouse suggesting that this relationship may also occur *in vivo* [21]. Scanning electron microscopy revealed the intimacy of the EC:Mf association in which the end of the Mf was embedded either onto or into or was burrowed beneath the endothelial cell. A structure on the anterior end was observed in this study that has been previously defined as a “hook” [31,32]. Although the function of this structure is not known, a role in contact between Mf and EC can be postulated and may have particular importance for initial adherence under flow conditions. In contrast to the intestinal epithelium-invading parasite *Trichuris muris*, *B*. *malayi* Mf do not possess grooves on their dorsal surface, and thus, do not appear to be adapted for migration with the full length of their body through a cellular layer [49]. The attachment of Mf to the cell surface is, however, of sufficient affinity to be maintained even in the presence of higher shear forces (1 dyne/cm^2^) or forces applied manually using a mechanical pipette-boy.

It remains uncertain how the circadian appearance of Mf in the peripheral blood is co-ordinated. The presence of melatonin and a lowered body temperature of 36°C are both associated with nocturnal physiology and several parasites have been shown to produce, as well as respond to, melatonin. *Trypanosoma cruzi* synthesises melatonin, which is important for its growth and transformation, and, host-derived melatonin increases parasitaemia and maturation of both *P*. *falciparum* and *P*. *chabaudi* [50,51]. Interestingly, the free-living nematode, *Caenorhabditis elegans*, is known to respond to melatonin via specific receptors; melatonin regulates locomotion and other homeostatic functions such as body phenotype and egg-laying in this nematode [52]. In our experimental *in vitro* model, however, neither melatonin nor lowered temperature altered the adherence of Mf to EC. One indication from our study is that reduced blood flow in the lung during vasoconstriction may control the appearance of nocturnally sub-periodic *B*. *malayi* Mf in the peripheral blood, as Mf were only able to adhere to EC under high flow conditions while under low flow conditions they did not bind.

Administration of DEC to infected individuals causes an immediate appearance of *B*. *malayi* Mf in the peripheral blood. The mechanism of this Mf release is not understood, however DEC is known to rapidly inhibit EC prostanoid metabolism [53]. Inhibition of prostacyclin and/or PGE_2_-mediated vasodilation may result in vasoconstriction, increased resistance and therefore decreased blood pressure may result in Mf release into the periphery [53]. This is corroborated by our observation that Mf bind EC at high, but not low, flow rates. At night, Mf are released when oxygen tension in the lungs is lower [4] and *in vitro* we have shown that *B*. *malayi* Mf are less able to bind EC under hypoxic conditions. Additionally, during general anesthesia, which in respect of lower temperature and oxygen tension is similar to sleep, we observed higher Mf release into the peripheral blood *in vivo*. Overall, Mf retention in the lungs is likely to depend on vascular tone, which can be altered by oxygen tension and temperature, both of which are altered during sleep.

Fixatives that cause the cross-linking of proteins and/ or polysaccharides led to enhanced Mf adherence to EC suggesting that Mf bind to one or more cell surface molecule(s), that may be both preserved and exposed during fixing [54]. Mf presence and their excretory-secretory products did not significantly alter the EC expression of the surface molecules tested [55,56]. Interestingly, Mf adherence was moderately enhanced by IFN-γ stimulation of EC, but not by TNF-α. Antibody blockade of molecules known to be upregulated by IFN-γ but not by TNF-α, such as HLA-A, HLA-B, HLA-C and HLA-DRα did not alter the level of Mf binding. However, a role for these molecules cannot be completely ruled out, as sites other than those bound by the monoclonal antibodies may be involved in Mf adherence. TNF-α stimulates EC surface expression of VCAM-1, P-selectin, E-selectin and ICAM-1 suggesting that these molecules are not involved in Mf binding, indeed, antibody blocking of ICAM-1 was ineffective in altering Mf adherence [33,35,36].

While fixation of HUVEC may cross-link or unmask Mf binding sites on the cell surface, IFN-γ also induces cytoskeletal rearrangements that could in turn reveal novel sites to which Mf adhere [57]. Interestingly, *S*. *mansoni* schistosomulae can adhere to bovine brain microvascular EC, and the number of schistosomulae attaching is reduced in the presence of cytochalasin D, an inhibitor of actin polymerization [58]. This indicates that cytoskeletal activity plays a role in the adherence of schistosomulae to EC, and it was hypothesised that an analogous situation may occur with *B*. *malayi* Mf. However, attempts in our laboratory to block cytoskeletal rearrangement of HUVEC using cytochalasin B did not inhibit Mf adherence to HUVEC.

The key observation of this study is that a major component of the early immune response, C3 and/or downstream components of the complement cascade, have a crucial role in adherence of Mf to live vascular EC. It is known that C3 can be deposited on the surface of live *B*. *malayi* Mf *in vitro*, and, in a mouse model, this C3 deposition was primarily mediated by MBL-A [41,59,60]. Notably, *L*. *carinii* (now *sigmodontis*) Mf, which showed little adherence to EC does not bind complement [60]. In *B*.*malayi* Mf, C3 deposition appears to be particularly abundant at the nematode tips, which may explain the fact that Mf mainly adhere to HUVEC with their ends. Furthermore, exsheathment of Mf reduced their ability to bind EC. The sheath is known to contain mostly protein and some 5% carbohydrate, the most abundant component of which is N-acetyl-glucosamine while exsheathed Mf do not bind lectin and do not have surface N-acetyl-glucosamine [61]. In our *in vitro* culture system as there is an absence of specific antibody, complement deposition on the surface of Mf is likely to be primarily via the MBL pathway, which can bind N-acetyl-glucosamine. Therefore in the absence of the sheath there will be little complement deposition via MBL.

HUVEC possess complement receptors, CR1 and CR4, on their surface. THP-1 possesses CR1, CR3 and CR4 while HEK cells do not have CR. CR1 ligates C3b with high affinity and iC3b with lower affinity, while CR4 ligates only iC3b [62,63,64]. Expression of the CR4 component, CD11c, was barely detectable on HUVEC in our study and is therefore unlikely to be important for Mf adherence [65]. In addition, when we blocked CR1 with an antibody known to prevent ligation of C3b, but not iC3b, there was little alteration in the level of Mf adherence [30,66,67]. This suggests that *B*. *malayi* Mf adherence to HUVEC may occur via the iC3b:CR1 axis [67]. Indeed, iC3b deposition has been shown on the surface of *Onchocerca volvulus* Mf following incubation with human serum, and factor H deposition (which can lead to iC3b formation) has been seen on the surface of both *O*. *volvulus* and *Loa loa* Mf [68,69]. Interestingly, while IFN-γ moderately enhanced Mf binding to HUVEC, it did not alter the level of CR1 or CR4 expression on EC, suggesting that IFN-γ-increased Mf adherence either occurs by augmenting the levels of complement components secreted by HUVEC themselves, or by, an as yet undefined mechanism. Intriguingly, levels of C3 and C4 follow a circadian pattern in humans and are at their lowest during the night between midnight and 6am which coincides with Mf release into the peripheral blood of the nocturnally periodic *B*. *malayi* Mf [70].

In conclusion, we report novel insights into the direct interaction of filarial Mf with vascular endothelial cells. Mf are known to display a circadian periodicity in their appearance in the peripheral blood which coincides with the mosquito vector’s feeding habits. Our studies reveal some parallels between the role of oxygen tension and vascular tone *in vivo* and the fluctuations in the ability of Mf to adhere to EC *in vitro*. In particular we show a central role for C3, a major component of the immune response, in this *in vitro* interaction. Thus our work may promote a greater understanding of the mechanism of this highly evolved adaptation for increasing effective transmission.

## Supporting information

S1 Supporting Information *B*. *malayi* Mf adhering to HUVEC with one or both ends. *B*. *malayi* Mf were co-cultured with HUVEC in medium supplemented with 10% human serum in static culture conditions. After 24 hours the co-culture was examined under a phase contrast microscope (magnification: 10 x). The video shows side-to-side movement of Mf bound to HUVEC with one or both ends. Non-binding Mf are also present.
(MOV)

S2 Supporting Information *B*. *malayi* Mf do not adhere to HUVEC under low flow rate conditions. *B*. *malayi* Mf were co-cultured with HUVEC in medium supplemented with 10% human serum at a flow rate of 0.1 dyne/cm^2^. Non-binding Mf are observed flowing past the HUVEC monolayer.
(MOV)

S3 Supporting Information Some *B*. *malayi* Mf do adhere to HUVEC under high flow rate conditions. *B*. *malayi* Mf were co-cultured with HUVEC in medium supplemented with 10% human serum at a flow rate 1.0 dyne/cm^2^. They adhere with one or both ends and can be seen moving from side to side while bound to HUVEC. Non-binding Mf are observed flowing past the HUVEC monolayer.
(MOV)

S4 Supporting Information A *B*.*malayi* Mf attempting to adhere to HUVEC under high flow conditions. Mf were co-cultured with HUVEC in medium supplemented with 10% human serum at a flow rate 1.0 dyne/cm^2^. Non-binding Mf are observed flowing past the HUVEC monolayer.
(MOV)

S5 Supporting Information *B*. *malayi* Mf adhere to THP-1. *B*. *malayi* Mf were co-cultured with PMA-stimulated THP-1 in medium supplemented with 10% human serum. After 24 hours the co-culture was examined under phase contrast microscopy (magnification: 10 x). The video shows side-to-side movement of Mf that are adhering to the THP-1 monolayer while other THP-1 cells are seen adhering to the entire Mf surface.
(MOV)

S6 Supporting Information *L*. *sigmodontis* Mf adhere to HUVEC in low numbers. *L*. *sigmodontis* Mf were co-cultured with HUVEC in medium supplemented with 10% human serum. After 24 hours the co-culture was examined under phase contrast microscopy (magnification: 10 x). The video shows side-to-side movement of some Mf bound to HUVEC with one end.
(MOV)

S7 Supporting Information Presence of intact bovine and human serum equally promotes *B*. *malayi* Mf adhere to HUVEC. *B*. *malayi* Mf were co-cultured with HUVEC in medium supplemented with 10% human or foetal bovine serum. After 24 hours the Mf adhering to the cell monolayer were counted. Data are shown as the mean ± standard deviation of three independent experiments.
(PPT)
