# Supplementary figures and images for: Brugia malayi microfilariae adhere to human vascular endothelial cells in a C3-dependent manner
Source: PLoS Negl Trop Dis. 2017 May 8;11(5):e0005592. doi: 10.1371/journal.pntd.0005592 (PMC5436873; doi:10.1371/journal.pntd.0005592)

## Slide 1
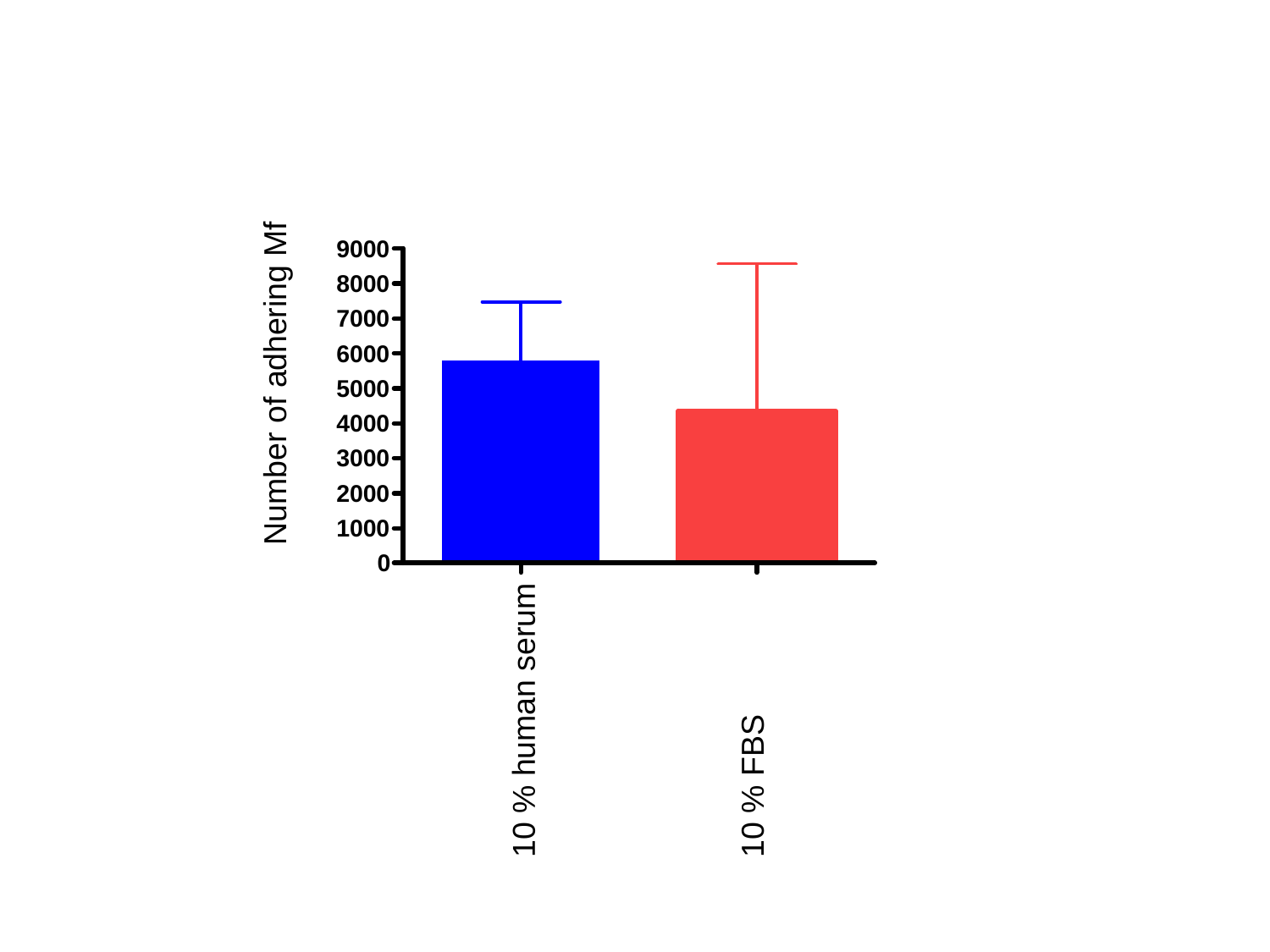

10 % human serum
10 % FBS
Number of adhering Mf

Supplement: S7 Supporting Information — B. malayi Mf were co-cultured with HUVEC in medium supplemented with 10% human or foetal bovine serum. After 24 hours the Mf adhering to the cell monolayer were counted. Data are shown as the mean ± standard deviation of three independent experiments. (PPT) [file pntd.0005592.s007.ppt]
